# Chromosomal requirements for formation of multivalent human nucleoli

**DOI:** 10.1101/gad.354012.126

**Published:** 2026-11-01

**Authors:** Krystyna Giemza, Hazel Mangan, Brian McStay

**Affiliations:** Centre for Chromosome Biology, College of Science and Engineering, University of Galway, Galway H91 W2TY, Ireland

**Keywords:** acrocentric chromosomes, nucleolar organizer region, nucleolus, ribosomal genes, chromosome engineering

## Abstract

Human nucleoli are multivalent, involving contributions from up to 10 NOR-bearing acrocentric chromosome p-arms. Precision megabase-scale chromosome engineering defines the requirements for this major genome organizational event. NOR deletions reveal that p-arm clustering can occur independent of rDNA. Deletion of all NOR-distal or NOR-proximal sequences individually have only a marginal effect on nucleolar association. Finally, deletion of an entire p-arm, while leaving centromere function intact, destroys the nucleolar association potential of that acrocentric. We propose that formation of multivalent nucleoli is not a nucleolar fusion event per se; rather it is driven by the surrounding chromosomal context of NORs.

## Abstract (precis)

In this study, Giemza et al. define the chromosomal domain features required for the association of acrocentric p-arms in multivalent nucleoli. While neither ribosomal DNA arrays nor nucleolar organizer regions (NORs) were found particularly essential for nucleolar association, the formation of multivalent nucleoli was driven by the propensity of p-arm self-association, providing an alternative to the prevailing “nucleolar fusion” theory of multivalent nucleolar organization.

Human chromosomes occupy distinct nonrandomly positioned territories and are folded into hierarchical domains to facilitate genome packaging and organization of functional compartments (Misteli 2020). Nucleoli, the most prominent functional compartments, form around arrays of ribosomal genes (rDNA) known as nucleolar organizer regions (NORs) located on the short p-arms of the five acrocentric chromosomes 13, 14, 15, 21, and 22 (McStay 2023).

In nontransformed human cell lines the default status of NORs is active as defined by the presence of UBF, a nucleolar-specific HMG-box DNA binding protein that binds extensively across the rDNA array (van Sluis et al. 2020). Upon postmetaphase reactivation of transcription, nucleoli with three recognizable compartments—fibrillar centers (FCs), dense fibrillar component (DFC), and granular component (GC)—reform (Mangan et al. 2017). FCs have a protein composition, including UBF, that has significant overlap with that of mitotic NORs. Each FC unit represents one or a few rDNA repeats with nascent transcripts extending into a surrounding DFC where early preribosomal RNA processing occurs. Late processing and ribosome subunit assembly are localized to the GC. While the biophysical property of key proteins has been offered as an explanation of how these three nucleolar subcompartments form, there is an absolute requirement for ongoing transcription (Shan et al. 2026).

Cells could in principle contain 10 nucleoli, yet most have between one and three, each involving multiple acrocentric p-arms, organized into discrete NOR territories with associated perinucleolar heterochromatin (PNH) (Mangan and McStay 2021; Leeke et al. 2025). We propose referring to these as “multivalent nucleoli,” in the sense that multiple acrocentric p-arms are involved in their formation, but no mechanism is implied. Thus, formation of multivalent nucleoli involves appropriate juxtaposition of multiple chromosome territories and hierarchical domains within them, representing a major genome organization event.

Human cells typically contain between 300 and 600 rDNA repeats, unevenly distributed among the five pairs of acrocentric p-arms (Stults et al. 2008; van Sluis et al. 2020; Potapova et al. 2025). NORs are bordered on their telomeric and centromeric sides by the distal junction (DJ) and proximal junction (PJ), respectively, shared among the acrocentrics (Fig. 1A; Floutsakou et al. 2013; van Sluis et al. 2019). The DJ (∼300 kb) is dominated by a large (>100 kb) inverted repeat, which encodes long noncoding RNAs (lncRNAs) required for NOR function. Three-dimensional immunoFISH with a DJ FISH probe has proved a reliable method for enumerating p-arms in each nucleolus. The arrival of telomere-to-telomere (T2T) genomes has delivered full sequences for acrocentric p-arms, heralding a new era of research into how they function (Nurk et al. 2022; Guarracino et al. 2023). They range in size from 10 to 17 Mb with NOR-distal and NOR-proximal sequences comprising multiple satellite DNA classes and segmental duplications (SDs).

**Figure 1.**
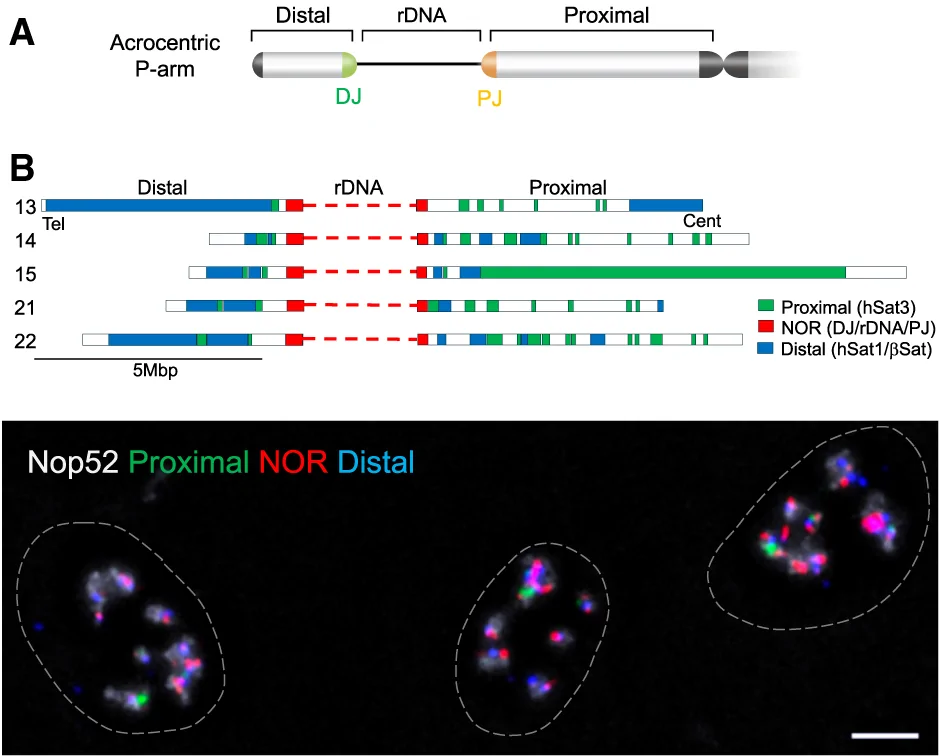
Acrocentric p-arms associate with nucleoli. (*A*) Organization of NORs on acrocentric p-arms, depicting the rDNA, distal and proximal regions, and distal (green) and proximal (orange) junctions (DJ and PJ). (*B*) Three-dimensional immunoFISH was performed on RPE1 cells with distal (blue), NOR (red), and proximal (green) p-arm regional probes and antibodies against Nop52. Probe compositions and the distribution of their target sequences on acrocentric p-arms in T2T CHM13 are shown *above*. Nuclear membranes are indicated by dashed white lines. Scale bars, 5 µm.

In the past, formation of multivalent nucleoli has been described as resulting from nucleolar fusion, implying that nucleoli on individual p-arms have a propensity to coalesce (Anastassova-Kristeva 1977; Mangan et al. 2017). Critically however, we cannot distinguish between cause and effect. It is equally plausible that acrocentric p-arms have a propensity to self-associate, with formation of multivalent nucleoli being a consequence. Here, we use CRISPR/Cas9-mediated megabase-scale precision chromosome engineering (PCE) (Essletzbichler et al. 2014) to determine the chromosomal requirements for the participation of acrocentric p-arms in multivalent nucleoli.

## Results and Discussion

### Sequences across acrocentric p-arms are nucleolar-associated

Utilizing a combination of rDNA and DJ probes, we had determined that in hTERT-RPE1 cells all 10 acrocentric p-arms are nucleolar-associated despite one of the chr22 haplotypes having little or no rDNA (van Sluis et al. 2020). To reveal the extent of acrocentric p-arm nucleolar association, we have designed three regional FISH probes that span the p-arms (Fig. 1B). An NOR probe (red) includes the rDNA array as well BACs encompassing both the DJ and PJ. Combined hSat1 and βSat probes (blue) recognize distal regions preferentially. Finally, hSat3 (green) was used to identify predominantly proximal regions. The distribution of sequences recognized by these regional probes on p-arms of the T2T CHM13 genome (Nurk et al. 2022) is shown. Three-dimensional immunoFISH using these p-arm regional probes was performed on RPE1 cells with an antibody to GC protein Nop52 (van Sluis et al. 2016) to visualize nucleoli (Fig. 1B; Supplemental Fig. S1). This revealed that all 10 acrocentric p-arms are intimately associated along their length with nucleoli, consistent with the PNH being largely composed of acrocentric p-arms. The weak hybridization signal from the distal (blue) probe that is not associated with nucleoli is due to pericentric satellites on individual metacentric chromosomes. Next, we performed PCE to elucidate the rules of engagement for acrocentric p-arms in multivalent nucleoli.

### Deletion of rDNA arrays from human acrocentrics in mouse A9 hybrids

To formally test the requirement for rDNA arrays, we sought to assess the nucleolar association potential of p-arms in which they had been deleted using CRISPR/Cas9. As DJ and PJ sequences are shared among the acrocentrics, we initially chose to perform the rDNA deletion on individual human chromosomes 14 and 15 (chr14 and chr15) held within mouse A9 monochromosomal hybrids A9-14 and A9-15, respectively (Cuthbert et al. 1995). The engineered chromosome was to be shuttled into human RPE1 cells by microcell-mediated chromosome transfer (MMCT) as previously described (Meguro-Horike and Horike 2015; Mangan and McStay 2021). MMCT involves production of microcells containing single chromosomes from A9 hybrid cells, fusing them with recipient RPE1 cells and selecting for recipient cells that have taken up the engineered human chromosome and no mouse chromosomes.

We designed a pair of gRNAs that would facilitate precise deletion of rDNA arrays from both chr14 and chr15 by CRISPR/Cas9. These gRNAs are located 804 bp and ∼6.6 kb distal and proximal to rDNA, respectively (Fig. 2A). By cotransfecting A9-14 and A9-15 cells with pairs of Cas9-gRNA RNPs and screening for novel DJ/PJ fusions, indicative of rDNA deletion, by PCR, we obtained two independent A9-14ΔhrDNA (#16 and #20) and two A9-15ΔhrDNA15 clones (#26 and #33) (Supplemental Fig. S2A). Sequencing revealed that DJ and PJ's had fused with the deletion of a few additional bases, or in the case of one of the A9-15 clones (#33), inclusion of 65 bp of mouse sequence (Supplemental Fig. S2A). Three-dimensional immunoFISH performed on the resulting clones confirmed the loss of human rDNA (Supplemental Fig. S2B).

**Figure 2.**
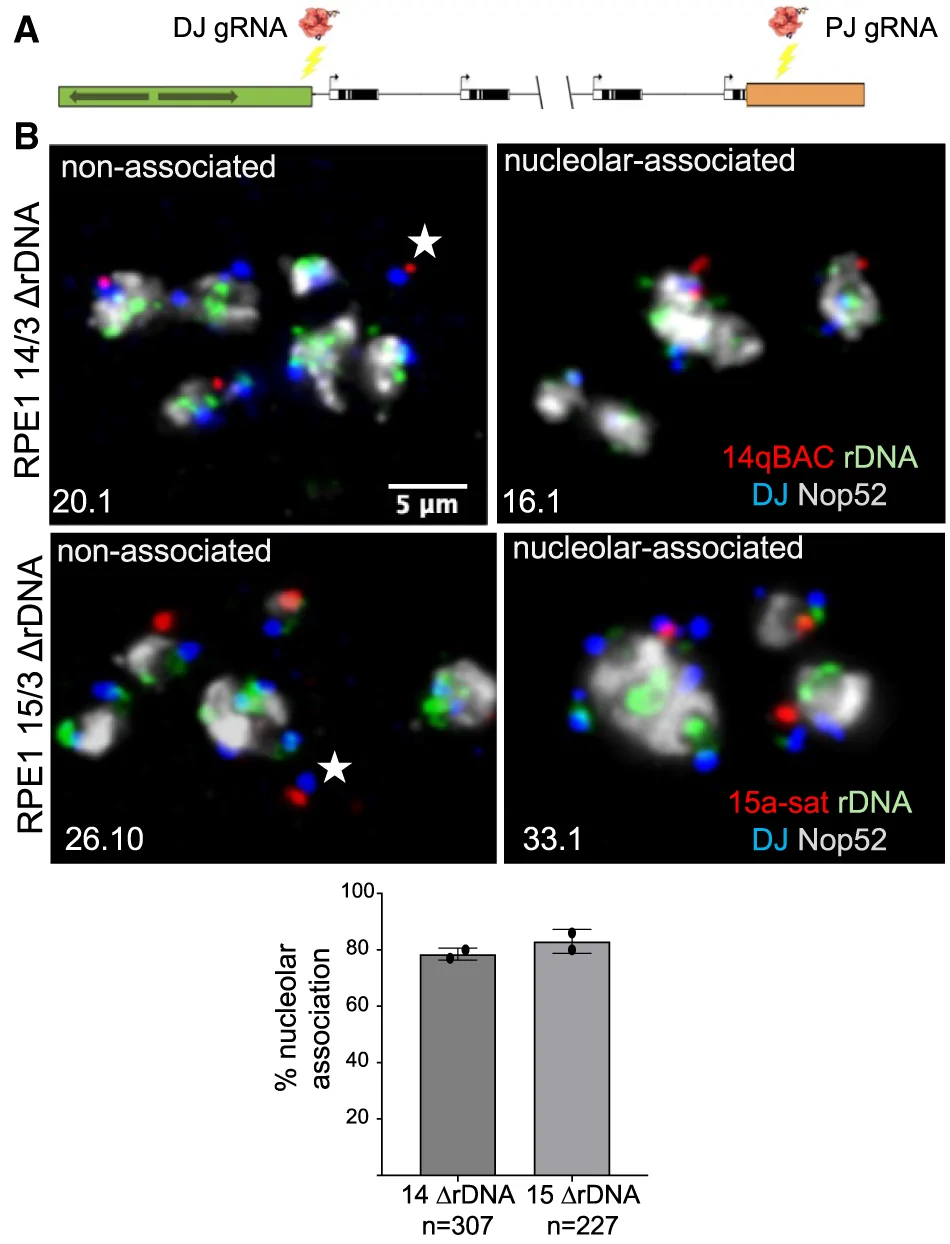
Deletion of rDNA from single human acrocentrics present in A9 monochromosomal hybrids. (*A*) Cartoon showing the locations of DJ and PJ gRNAs. (*B*) Three-dimensional immunoFISH on RPE1 14/3 ΔrDNA (*top* panels) and RPE1 15/3 ΔrDNA (*bottom* panels) containing 14ΔrDNA and 15ΔrDNA chromosomes from A9-14 and A9-15, respectively. Each panel represents a single nucleus. FISH probes included rDNA, DJ, and either a chr14 q-arm BAC or α-sat15 and Nop52 antibodies. Representative images of cells in which the transferred chromosome was nucleolar-associated or nonassociated (indicated by an asterisk) are shown, with the clone identities indicated. Quantitation is shown *below*. Bars represent the mean ± standard deviation (SD) across both clones. (*n*) Number of cells analyzed.

### rDNA arrays are not required for association of acrocentric p-arms with multivalent human nucleoli

To test for the impact of rDNA deletion on nucleolar association potential in human cells, the engineered human acrocentrics were transferred into human RPE1 cells by MMCT as previously described (Mangan and McStay 2021). Successful chromosome transfer and the absence of carried over mouse chromosomes was confirmed by a combination of metaphase FISH (m-FISH) performed on spreads and STR (short tandem repeat) analysis (Supplemental Fig. S2C,D). We isolated two RPE1 clones containing an rDNA deleted chr14, one from each of the independent A9-14 ΔhrDNA clones (#16.1 and #20.1). Additionally, we isolated four RPE1 clones containing an rDNA deleted chr15, three from one A9-15 ΔhrDNA #26 (26.8, 26.9, 26.10) and one from #33 (33.1) (Supplemental Fig. S2C).

To assess the nucleolar association potential of the engineered chromosome, we performed 3D immunoFISH on interphase cells, combining a chr15-specific α-satellite (α-sat15) centromere probe, DJ and human rDNA probes (Fig. 2B). ΔhrDNA15 could be distinguished from the endogenous chr15s by the absence of rDNA. Nucleoli were identified using antibodies to Nop52. To visualize ΔhrDNA14, we replaced a centromeric probe with a BAC that maps immediately proximal to centromere on the q-arm of chr14 (van Sluis et al. 2020). This was required as a chr14 centromere probe also recognizes chr22, complicating the analysis. Combining the results from independent clones, we determined that in 78.5% and 81.5% of cells, the ΔhrDNA14 and ΔhrDNA15, respectively, were observed to be nucleolar-associated (Fig. 2B).

Next, we turned our attention to deleting endogenous rDNA arrays from acrocentrics present within standard RPE1 cells. While initially hesitant to do this, as the DJ and PJ gRNA sequences are present on all acrocentrics, we were able to isolate a clone, RPE1-ΔrDNA13, in which the rDNA arrays present on both chr13 haplotypes had been deleted. rDNA deletion was confirmed by PCR, which amplified the novel DJ/PJ junctions. Sequencing of eight independent cloned PCR products revealed that both haplotypes have identical precise DJ/PJ fusions (Supplemental Fig. S3A). Loss of rDNA was further confirmed by m-FISH (Fig. 3A; Supplemental Fig. S3B). This also confirmed that the remaining acrocentrics retained their normal rDNA content.

**Figure 3.**
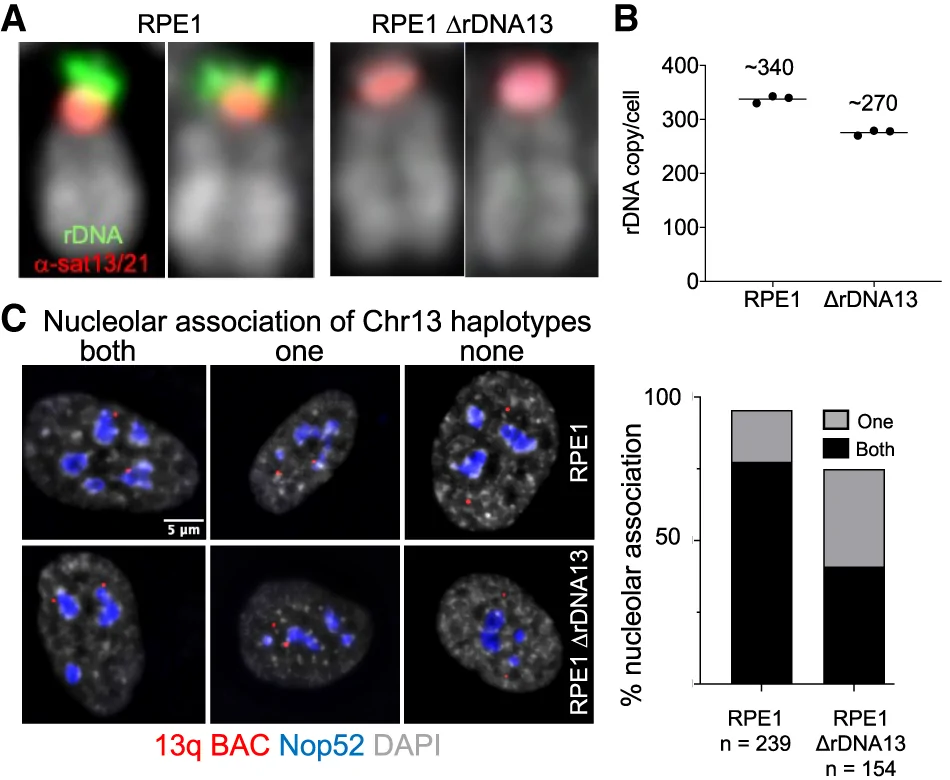
Deletion of endogenous rDNA arrays in RPE1 cells. (*A*) M-FISH was performed on normal RPE1 cells and RPE1-ΔrDNA13 cells with α-sat13/21, α-sat14/22, and rDNA FISH probes. Isolated chr13 pairs from each are shown. The full spreads are shown in Supplemental Figure S3B. (*B*) rDNA repeat copy numbers in RPE1 and RPE1-ΔrDNA13 cells were determined by digital PCR, performed in triplicate. The average repeat number for each cell type is shown *above*. (*C*) Three-dimensional immunoFISH on RPE1 and RPE1-ΔrDNA13 with a 13q BAC FISH probe and Nop52 antibodies. Representative images for cells in which both, one, or no chr13s are associated with nucleoli are shown. Quantitation is shown on the *right*. (*n*) Number of cells analyzed.

Previous analysis had revealed that in RPE1 cells both chr13s have moderate sized rDNA arrays (van Sluis et al. 2020). Thus, we expected an observable decrease in rDNA copy number. In line with expectation, digital PCR confirms that the rDNA copy number per cell drops from ∼340 repeats in RPE1 cells to ∼270 repeats in RPE1-ΔrDNA13 cells, a drop of 20% (Fig. 3B).

To visualize chr13s in RPE1-ΔrDNA13 interphase cells, we combined a 13q centromere-proximal BAC probe (van Sluis et al. 2020) with Nop52 antibodies (Fig. 3C). In 75% of RPE1-ΔrDNA13 cells, we observed that one or both chr13s are nucleolar-associated. This compares to 95.7% in standard RPE1 cells. We conclude that while rDNA arrays are not essential for participation of acrocentric p-arms in multivalent nucleoli they likely contribute. This is further supported by our previous studies with neoNORs, synthetic rDNA arrays on metacentric chromosomes, that can associate with endogenous multivalent nucleoli (Grob et al. 2014).

### Role of NOR-distal and NOR-proximal sequences

Then we turned our attention to the role of NOR-distal and NOR-proximal p-arm regions in formation of multivalent nucleoli. Initially we focused on the NOR-distal region of chr15. We combined the distal gRNA used for rDNA deletion with a gRNA positioned ∼32 kb from the telomere of chr15 (Fig. 4A). Notably, this telomeric gRNA appears unique to chr15 and is present within the T2T CHM13 genome (Nurk et al. 2022) as well as our A9-15 p-arm sequences (data not shown). This gRNA pair was used to generate RPE1-15Δdistal, in which the NOR-distal region of one chr15 had been deleted, resulting in the telomere now being positioned ∼32 kb from the rDNA array. PCR and DNA sequencing confirmed the presence of this novel junction (Supplemental Fig. S4A). The two chr15 haplotypes of RPE1 cells can be distinguished based on their rDNA content and the extent of centromeric α-sat DNA (Supplemental Fig. S5). One haplotype, labeled here as Hap1, has a relatively small rDNA array and a large α-sat array, while the other (Hap2) has a large rDNA array and a low amount of α-sat. The NORs on both are active as determined by the presence of UBF on metaphase chromosomes (van Sluis et al. 2020). ImmunoFISH performed on metaphase spreads from RPE1-15Δdistal revealed that the deletion had occurred on the high rDNA/low α-sat Hap2 and revealed the presence of UBF on the deleted chromosome (Fig. 4B). Thus, we can conclude that the rDNA array on the deleted chromosome is active, despite the absence of a DJ and the positioning of a telomere immediately distal.

**Figure 4.**
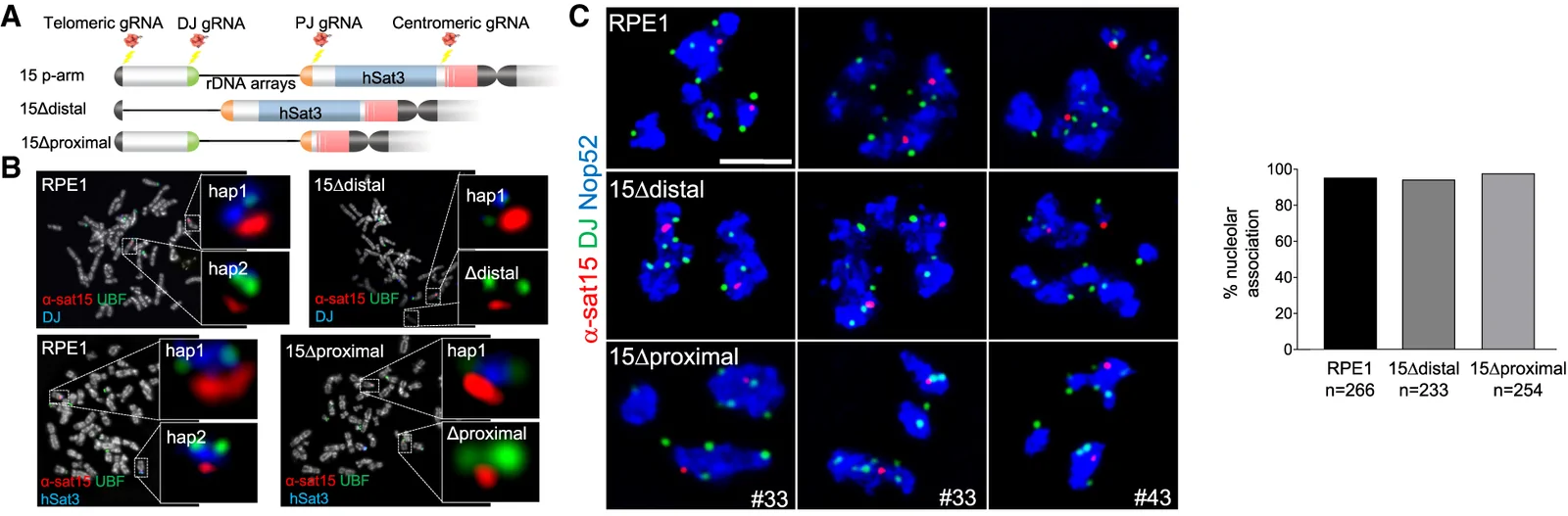
Consequences of deleting NOR-distal or NOR-proximal regions. (*A*) Cartoon showing the CRISPR-mediated distal and proximal deletion strategies and location of relevant gRNAs. (*B*, *top* panels) ImmunoFISH of metaphase spreads from RPE1 and RPE1-15Δdistal cells probed with α-sat15 and DJ FISH probes and hUBF antibodies. (*Bottom* panels) ImmunoFISH on metaphase spreads from RPE1 and RPE1-15Δproximal cells probed with α-sat15 and hSat3 FISH probes and hUBF antibodies. In each case, the p-arm of both chr15 haplotypes are shown enlarged. (*C*) Three-dimensional immunoFISH on RPE1 (*top* panels), RPE1-15Δdistal (*middle* panels), and RPE1-15Δproximal (*bottom* panels) cells with DJ and α-sat15 FISH probes and Nop52 antibodies. A single nucleus is shown in each panel, and the clone names for both RPE1-15Δproximal clones are indicated. Scale bar, 5 µm. Quantitation of nucleolar association is shown *below*. (*n*) Number of cells analyzed. Note that for RPE1-15Δproximal cells, we have pooled data from clones #33 and #43.

To determine whether this distal deletion impacted on the involvement of this chromosome in multivalent nucleoli, RPE1 and RPE1-15Δdistal interphase cells were probed with α-sat15 and DJ FISH probes combined with Nop52 antibodies (Fig. 4C). In standard RPE1 cells, we observed that both chr15s are associated with a multivalent nucleolus, determined by the presence of multiple DJ signals, in 95% of cells. In RPE1-15Δdistal we observe that both 15s are associated with a multivalent nucleolus in 93% of cells. We can now conclude that the telomere to rDNA chromosome interval, including the DJ, is not essential for the participation of an acrocentric in multivalent nucleoli.

To assess the role of NOR-proximal region of p-arms in formation of multivalent nucleoli, we sought to delete the rDNA to centromere interval from chr15 within RPE1 cells. We designed a chr15-specific gRNA that is positioned on the p-arm ∼1.7 Mb from the centromere in T2T CHM13 (Fig. 4A). This gRNA was similarly positioned on 15p in A9-15. The chr15 centromeric gRNA was combined with the PJ gRNA used above to delete rDNA. This allowed us to generate two independent RPE1-15Δproximal clones (#33 and #43). Probing metaphase spreads from RPE1-15Δproximal clones revealed that in both, the high rDNA/low α-sat15 Hap2 had been targeted (Fig. 4B). Sanger sequencing of PCR products from independent clones confirmed they contained the novel junction (Supplemental Fig. S4B). The deleted chromosomes were further shown to lack the major hSat3 array normally located proximal to rDNA. Finally, we could confirm that in 95% of spreads, the rDNA array remained UBF-loaded, and thus active, despite proximity to the centromere.

Three-dimensional immunoFISH reveals that in WT RPE1 and RPE1 15Δproximal cells, both 15 p-arms are found associated with a multivalent nucleolus (multiple DJ signals) in 95% and 98% of cells, respectively. Thus, we conclude that the rDNA to centromere interval of chr15 is not essential for the participation of an acrocentric in multivalent nucleoli (Fig. 4C).

We have now demonstrated that neither NOR-distal or NOR-proximal regions are individually essential for inclusion of acrocentric p-arms in multivalent nucleoli. Combining these findings with the lack of an rDNA requirement, demonstrated above, points to two contrasting hypotheses. The first posits that acrocentric p-arms have redundant features, both NOR-distal and NOR-proximal, capable of promoting their association. An alternative hypothesis is that the signal for association lies elsewhere on the acrocentric chromosome. Published observations that centromeres associate with nucleoli, particularly in human ESCs, point to the possibility that “specialised” acrocentric centromeres may be involved (Rodrigues et al. 2023; Yeo et al. 2026). To distinguish between these competing models, we next sought to delete an entire acrocentric p-arm, while leaving its centromere intact.

### Acrocentric p-arm deletion

Deletion of an entire acrocentric p-arm in RPE1 cells was achieved by combining the chr15-specific subtelomeric and centromeric gRNAs utilized above (Fig. 5A). We isolated a clone, RPE1-15ΔP, in which the p-arm of the low rDNA/high α-sat15 Hap1 had been deleted. DNA sequencing showed the presence of unique telomere/centromere fusion (Supplemental Fig. S6A). Further confirmation came from m-FISH, which reveals that hSat3, rDNA, DJs, and PJs had been deleted in Hap1ΔP (Fig. 5B; Supplemental Fig. S6B). Additionally, a telomere FISH signal now abuts the centromere on Hap1ΔP (Fig. 5C; Supplemental Fig. S6C). We have not observed any chromosomal instability in this clone, indicating that juxtaposition of the telomere and centromere has no measurable impact on either of their functions.

**Figure 5.**
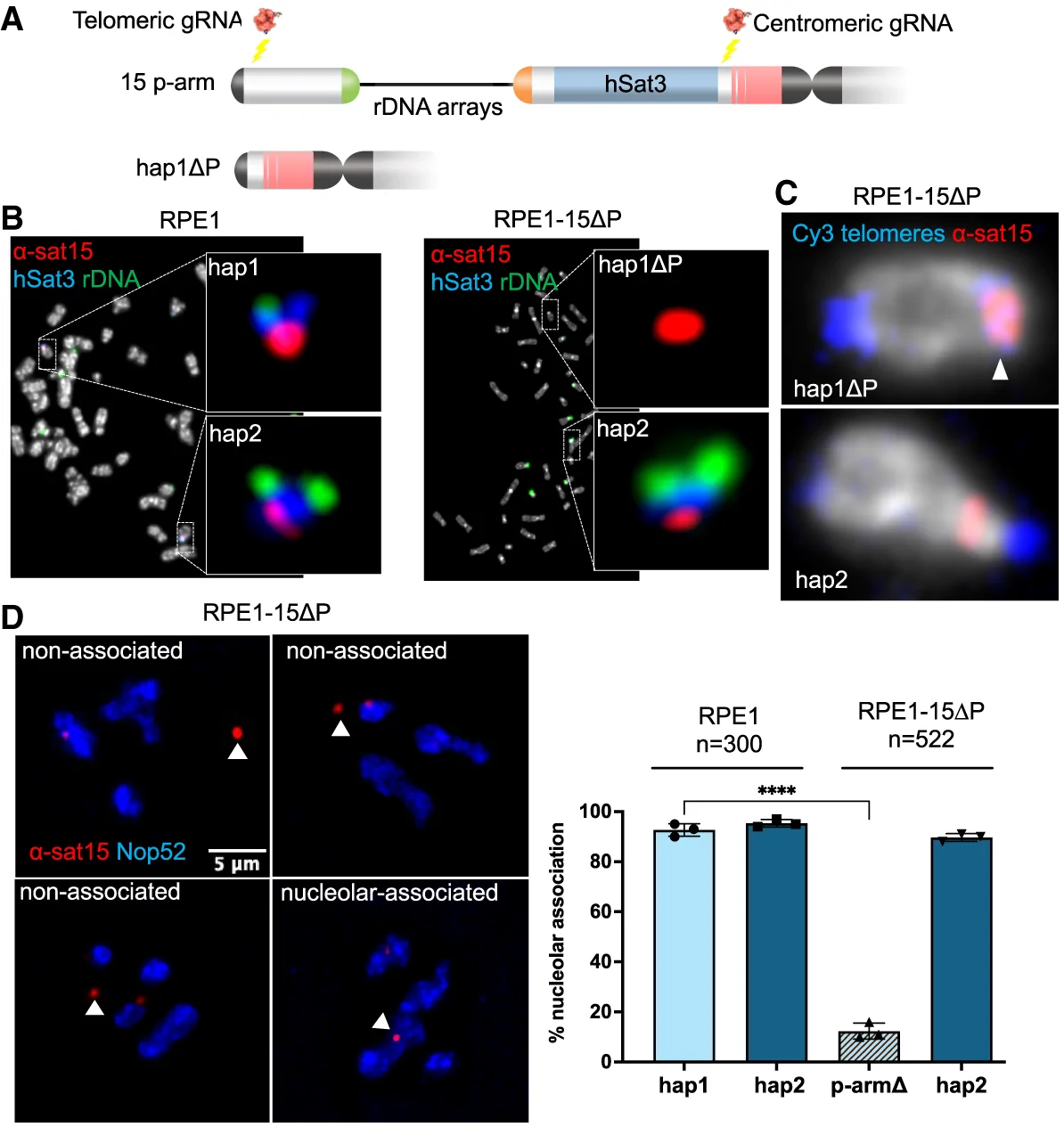
Deletion of full acrocentric p-arm. (*A*) Cartoon showing the CRISPR-mediated p-arm deletion strategy and location of relevant gRNAs. (*B*) M-FISH on RPE1 and RPE1-15ΔP with α-sat15, hSat3, and rDNA probes. Both chr15 haplotypes for each cell type are enlarged. (*C*) FISH on RPE1-15ΔP metaphase spreads with a telomere (Cy3-labeled) and α-sat15 probes. Both chr15 haplotypes are shown. The arrowhead indicates juxtaposed telomere and centromere signals on Hap1ΔP. The full metaphase spread is shown in Supplemental Figure S6C. (*D*) Three-dimensional immunoFISH on RPE1-15ΔP cells with α-sat15 and Nop52 antibodies. Each panel depicts a single nucleus, and the position of the Hap1ΔP in each is indicated with an arrowhead. Quantitation of the nucleolar association for each chr15 haplotype is shown on the *right*. (*n*) Number of cells analyzed.

To address the impact of p-arm deletion on the chromosome positioning, normal RPE1 and RPE1-15ΔP cells were probed using α-sat15 and a Nop52 antibody. In normal RPE1 cells α-sat15 signals from Hap1 and Hap2 contact nucleoli in 95% and 98% of cells, respectively (Fig. 5D). In contrast, within RPE1-15ΔP cells the Hap1ΔP centromere is dramatically repositioned, abutting nucleoli in only 12% of cells. The positioning of chr15 Hap2 remains unaltered. Thus, we conclude that chromosomal features that facilitate gathering of acrocentric chromosomes leading to the formation of multivalent nucleoli are restricted to acrocentric p-arms and do not involve either specialized acrocentric centromeres or q-arms.

Combined, the results from our PCE experiments provide compelling evidence that formation of multivalent nucleoli is primarily driven by the propensity of acrocentric p-arms to self-associate. Moreover, the chromosomal domains underlying this are redundant and distributed along the length of acrocentric p-arms.

Other than rDNA, the DJ and PJ are the only sequences that are shared in sequence and relative position among all five acrocentrics, yet neither of these is essential for acrocentric association. The distal deletion that we generated removes most of the DJ. As systemic depletion of DJ encoded lncRNAs negatively impacts ribosome biogenesis (van Sluis et al. 2019; Ataei et al. 2025), we were surprised that rDNA, now abutting the telomere on the deleted chromosome, remained active.

NOR-distal and NOR-proximal regions of acrocentric p-arms are composed largely of a number of satellite DNA classes and segmental duplications in various combinations and lengths (Nurk et al. 2022; Guarracino et al. 2023; McStay 2023). Thus, apart from rDNA, PJs, and DJs, acrocentric p-arms are more thematically similar rather than displaying high sequence similarity along their lengths, heterochromatin being the obvious theme. While the primary aim of this work was identifying the underlying DNA that drives acrocentric associations, our findings point to a role for heterochromatin. The proliferation marker Ki-67, which spatially organizes heterochromatin in early G1, may be involved (Booth et al. 2014; Schichler et al. 2026).

While a number of vertebrates, for example *Xenopus laevis* and rat kangaroo (*Potorous tridactylus*) have a single NOR per chromosome set, most mammals have multiple NORs. Not only does this provide redundancy, as evidenced by the lack of phenotype observed in individuals carrying a Robertsonian translocation (Guarracino et al. 2023; de Lima et al. 2025), it also provides greater opportunity for fixing chromosomal position within the nucleus. Thus, in humans, the self-association of acrocentric p-arms to form multivalent nucleoli provides a major positional cue for at least the 10 acrocentric chromosomes. Three-dimensional immunoFISH with a DJ probe, acrocentric paints and Nop52 antibodies, demonstrates that acrocentric q-arms make minimal contact with the nuclear lamina (Supplemental Fig. S7). As acrocentric associations are largely independent of rDNA, the correct positioning of acrocentrics with low or no rDNA, is ensured. This is nicely illustrated in the primary cell line CCD-1079Sk, fibroblasts that were isolated from the skin of a normal male neonate donor. Within these cells, four of the 10 acrocentrics lack detectable rDNA yet are associated with multivalent nucleoli (van Sluis et al. 2020).

Previously we have argued that acrocentric p-arms are dedicated to ribosome biogenesis, with the chromosomal context of rDNA arrays ensuring both their genetic isolation and genomic stability (McStay 2023). Additionally, the p-arm derived PNH may provide physical separation of nucleoli from the remaining nucleoplasm. To these functions we suggest including positional cues for acrocentric chromosomes that are independent of their nucleolar roles.

## Materials and methods

### Cell culture

A9 hybrids and hTERT-RPE1 cells were maintained as previously described (van Sluis et al. 2019). The RPE1 cells used as recipients for MMCT were a hygromycin resistance knockout version of the original RPE1 cells (Mangan and McStay 2021). CRISPR-based deletions within RPE1 cells used a version of RPE1 cells in which both puromycin and hygromycin resistance markers had been knocked out. MMCT was carried out as previously described (Meguro-Horike and Horike 2015; Mangan and McStay 2021).

### Precision chromosome engineering

A near full T2T genome for RPE1 cells has recently been determined (Volpe et al. 2025). However, as the rDNA arrays and much of the acrocentric p-arms are missing, we utilized T2T CHM13 (Nurk et al. 2022) and our own data from A9 hybrids to design gRNAs (van Sluis et al. 2019). Production of gRNAs and CRISPR genome editing were performed as previously described (Mangan and McStay 2021). Briefly, 4 µg of each gRNA was precomplexed with 8.2 µg of Alt-R S.p. Cas9 nuclease V3 (Integrated DNA Technologies) for 10 min at room temperature. RNPs were combined and coelectroporated with 500 ng of puromycin mRNA into 100,000 cells using three pulses of 1400 V for 10 msec for A9 cells and two pulses of 1350 V for 20 msec for RPE1 cells using a Neon transfection system (Thermo Fisher Scientific). Two hours after transfection, cells were selected with puromycin (Sigma) for 48 h at 2 µg/mL for RPE1 cells or at 10 µg/mL for A9 cells. Cells were seeded for single-cell colonies. Following 14 days, single-cell colonies were picked into 24 well plates. Seven days later, individual colonies were screened using Phire direct PCR master mix (Thermo Fisher Scientific) for the presence of novel junctions. Oligonucleotide sequences used in gRNA production and PCR are listed in Supplemental Table S1.

### Fluorescent in situ hybridization (FISH) on cells and chromosome spreads

For description of all FISH probes, see the Supplemental Material. Three-dimensional immunoFISH and m-FISH were performed as previously described (Mais et al. 2005; van Sluis et al. 2016). For m-FISH, fixed cells were dropped onto prechilled glass slides. For immunofluorescence on metaphase spreads, nonaged slides were fixed in 1% (w/v) paraformaldehyde (PFA) in 1× PBS for 10 min and then subjected to FISH. UBF was immunostained after hybridization, followed by three 15 min PBS washes. Slides were incubated with secondary antibodies (Jackson Immuno Research) for 1 h at 37°C, followed by three 15 min PBS washes. Slides were mounted in VectorShield (Vector Laboratories) with or without DAPI. All primary and secondary antibodies are listed in Supplemental Table S2. Telomere FISH is described in the Supplemental Material.

### Microscopy

All images were captured using a Leica DMi8 microscope as previously described (van Sluis et al. 2019). To assess nucleolar association, α-sat15 was used for chr15. For chr13 and chr14, BAC probes were used (Supplemental Table S1). Distances to nucleolar boundary were measured from maximum intensity projections. Acrocentric chromosomes were scored as nucleolar-associated if α-sat/BAC signal overlapped with, or was positioned within 0.5 µm of the nucleolar boundary. For RPE1 cells, nucleolar boundary was identified using an antibody to the granular component protein Nop52. For A9 hybrids, nucleolar boundary was identified with either Treacle or Nucleolin, as the human Nop52 antibody does not recognize mouse Nop52.

## Supplemental Material

Supplement 1
