# Supplementary material for: Chromosomal requirements for formation of multivalent human nucleoli
Source: Genes Dev. 2026 Nov 1;40(21-22):1818–24. doi: 10.1101/gad.354012.126 (PMC13726607; doi:10.1101/gad.354012.126)
Supplement: Supplementary file 1 [file Supplemental_Material.pdf]

Chromosomal requirements for formation of multivalent human nucleoli

Krystyna Giemza, Hazel Mangan and Brian McStay

List of Supplemental Material

Supplemental Materials and Methods

Supplemental References

Supplemental Figures S1-S7

Supplemental Table S1-S2

Supplemental Material and Methods

Preparation of FISH probes

DNA probes including, human rDNA, DJ, PJ, q-arm BACs and α-sat were prepared as previously described (Floutsakou et al. 2013; van Sluis et al. 2019). A series of FISH probes spanning the p-arms including hSat1, hSat3 and β-Sat were developed by PCR using genomic DNA from RPE1 cells and primers listed in Supplemental Table S1. Probes were directly labelled by nick translation using Green 496 dUTP, Red 580 dUTP, or Far Red 650 dUTP (Enzo).

Identification of chromosomes on chromosome spreads was done using paints for all acrocentrics prepared by Inter-Alu PCR (Liu et al. 1993). DNA from mono-chromosomal hybrids was used as template and amplified by KAPA HiFi DNA polymerase (Kapa Biosystems) using Inter-Alu primers (Supplemental Table S1). Chromosome paints were biotin-labelled using a BioPrime kit (Invitrogen) and visualized using Cy3 streptavidin (Rockland) (Mangan and McStay 2021).

Telomere FISH

M-FISH slides were washed with two 15-min 1xPBS washes and fixed in 4% (w/v) PFA for 2 min, followed by three 15-min washes. Slides were digested with pre-warmed pepsin solution (0.2 w/w% pepsin with 10 mM of HCl) for 10 min at 37°C, washed and refixed. Following three 15-min 1xPBS washes, slides were dehydrated in ethanol series and air-dried. Hybridization was performed using 200 nM Cy3-conjugated PNA telomere probe (Eurogentec) in 100 µL of hybridization buffer comprising 10 mM Tris (pH 7.4), 25 mM $MgCl_2$, 70% deionized formamide and 0.5% non-fat dry milk. For identification of chr15s in RPE1 cells, 5 µl of α-sat FISH probe was spiked into the hybridization mix. The probe was denatured at 80°C for 3 min. Slides were incubated in a dark humidified chamber for 1 h at room temperature, washed twice with washing buffer (70% formamide, 10 mM Tris; pH7.2, 1% BSA), followed by three 15-min 1xPBS washes. Slides were dehydrated as before, air-dried and mounted in Vectashield with DAPI.

Digital PCR (dPCR)

Genomic DNA was analysed by dPCR using TaqMan MGB probes with a QuantStudio Absolute Q Instrument and QuantStudio Absolute Q Software (Thermo Fisher Scientific). The rDNA probe spans the internal transcribed spacer 1 (ITS1) - 5.8S rRNA junction. As the PJ is conserved across the five human acrocentric chromosomes and is present in 10 copies per RPE1 cell (Floutsakou et al. 2013), a PJ probe provides an internal standard. Further details on dPCR will be published elsewhere.

Supplemental References

Floutsakou I, Agrawal S, Nguyen TT, Seoighe C, Ganley AR, McStay B. 2013. The shared genomic architecture of human nucleolar organizer regions. *Genome Res* **23**: 2003-2012.

Liu P, Siciliano J, Seong D, Craig J, Zhao Y, de Jong PJ, Siciliano MJ. 1993. Dual Alu polymerase chain reaction primers and conditions for isolation of human chromosome painting probes from hybrid cells. *Cancer Genet Cytogenet* **65**: 93-99.

Mangan H, McStay B. 2021. Human nucleoli comprise multiple constrained territories, tethered to individual chromosomes. *Genes Dev* **35:** 483-488.

van Sluis M, Gailin MO, McCarter JGW, Mangan H, Grob A, McStay B. 2019. Human NORs, comprising rDNA arrays and functionally conserved distal elements, are located within dynamic chromosomal regions. *Genes Dev* **33**: 1688-1701.

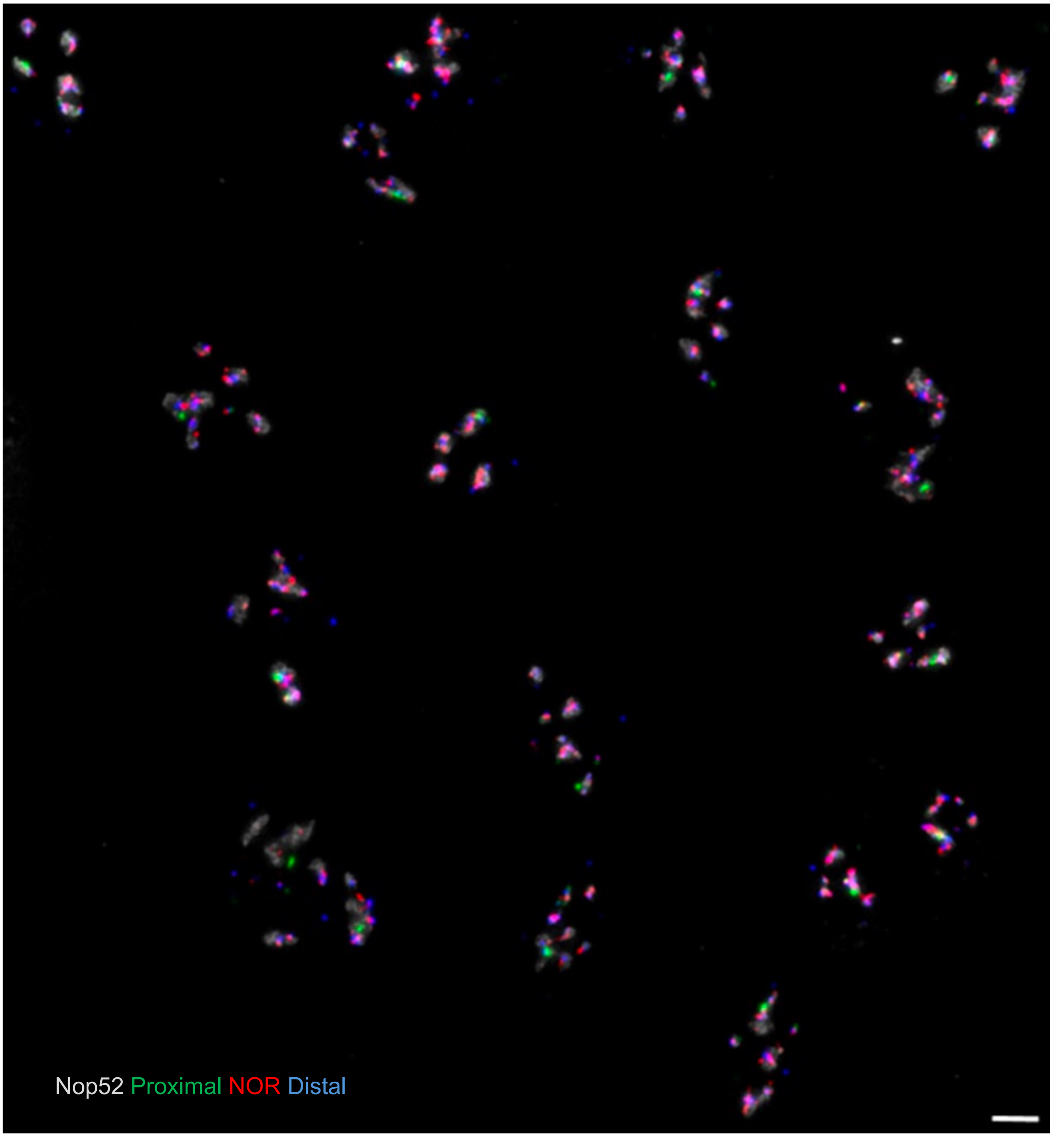

Supplemental Fig. S1 Acrocentric p-arms associate with nucleoli. 3D-immunoFISH was performed on RPE1 cells with distal, NOR and proximal p-arm regional probes and antibodies against Nop52. Scale bars represent 5µm. See Fig.1B for further details on probes.

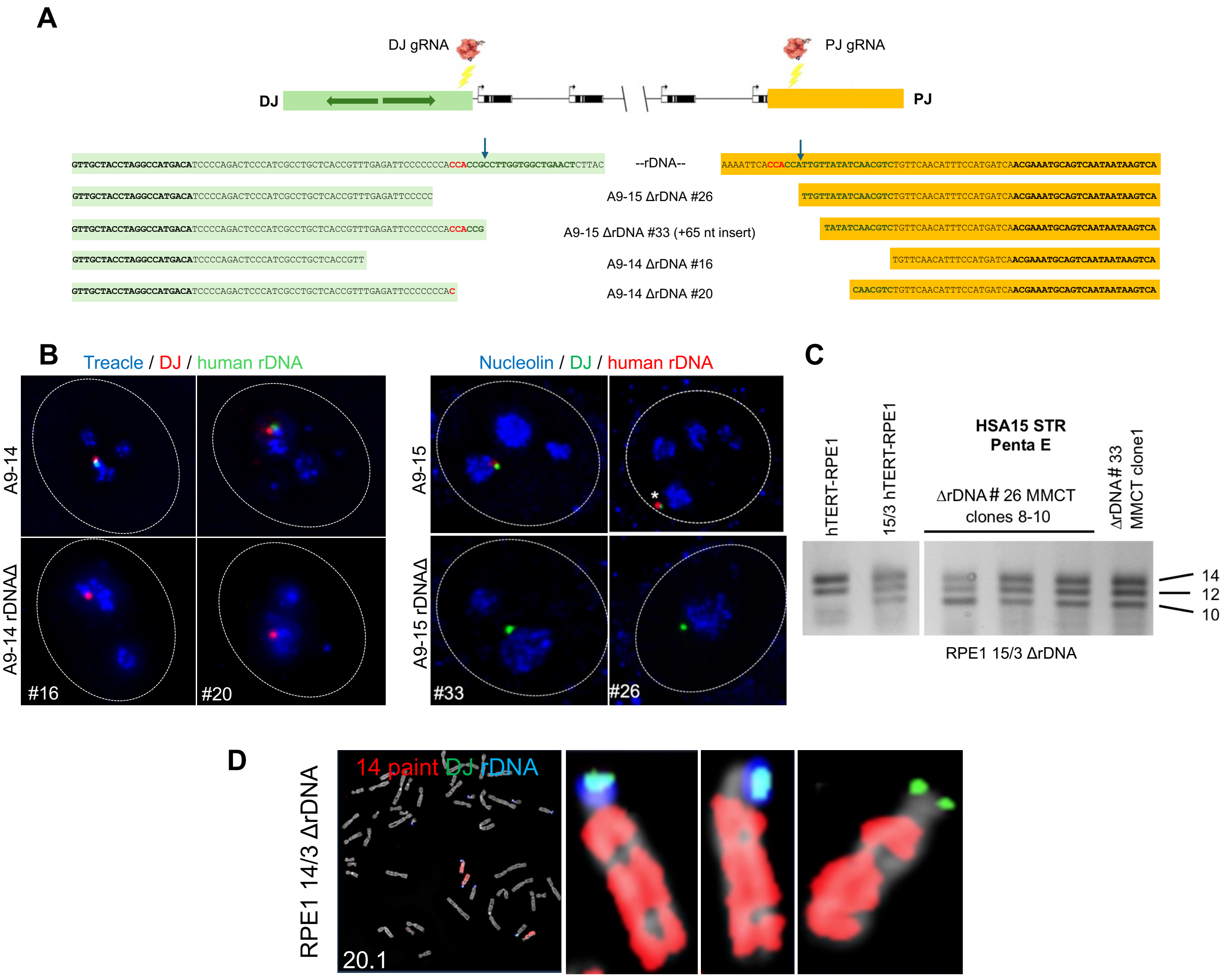

Supplemental Fig. S2 Deletion of rDNA arrays on human acrocentrics in A9-15 and A9-14 hybrids. (A) A cartoon depicting the relative position of DJ and PJ gRNAs, under which is shown the sequences surrounding both gRNAs. The screening primers (bold) are at the start and end of the DJ and PJ sequences respectively. gRNA sequences (green) and PAM sites (red) are indicated. The fusion products, as determined by sanger sequencing of cloned PCR products, for A9-15ΔrDNA and A9-14ΔrDNA clones are indicated below. (B) 3D-immunoFISH on A9-14, A9-15, A9-14ΔrDNA and A9-15ΔrDNA cells using DJ and human rDNA FISH probes and either Treacle or Nucleolin antibodies to visualise endogenous nucleoli in A9 mouse cells. (C) Penta E STR analysis performed on RPE1 cells and RPE1 15/3 cells which contain the intact chr15 from A9-15 cells (left panel) and both RPE1 15/3ΔrDNA clones #26 and #33(right panel). The PentaE 10 repeat haplotype is derived from A9-15 cells and absent in standard RPE1 cells that contain 12 and 14 repeat haplotypes. (D) M-FISH on RPE1 14/3 ΔrDNA with rDNA and DJ FISH probes and a chr14 paint. The three chr14s present in this cell line are shown on the right. Note the absence of rDNA on the transferred chr14 (14ΔrDNA, rightmost panel).

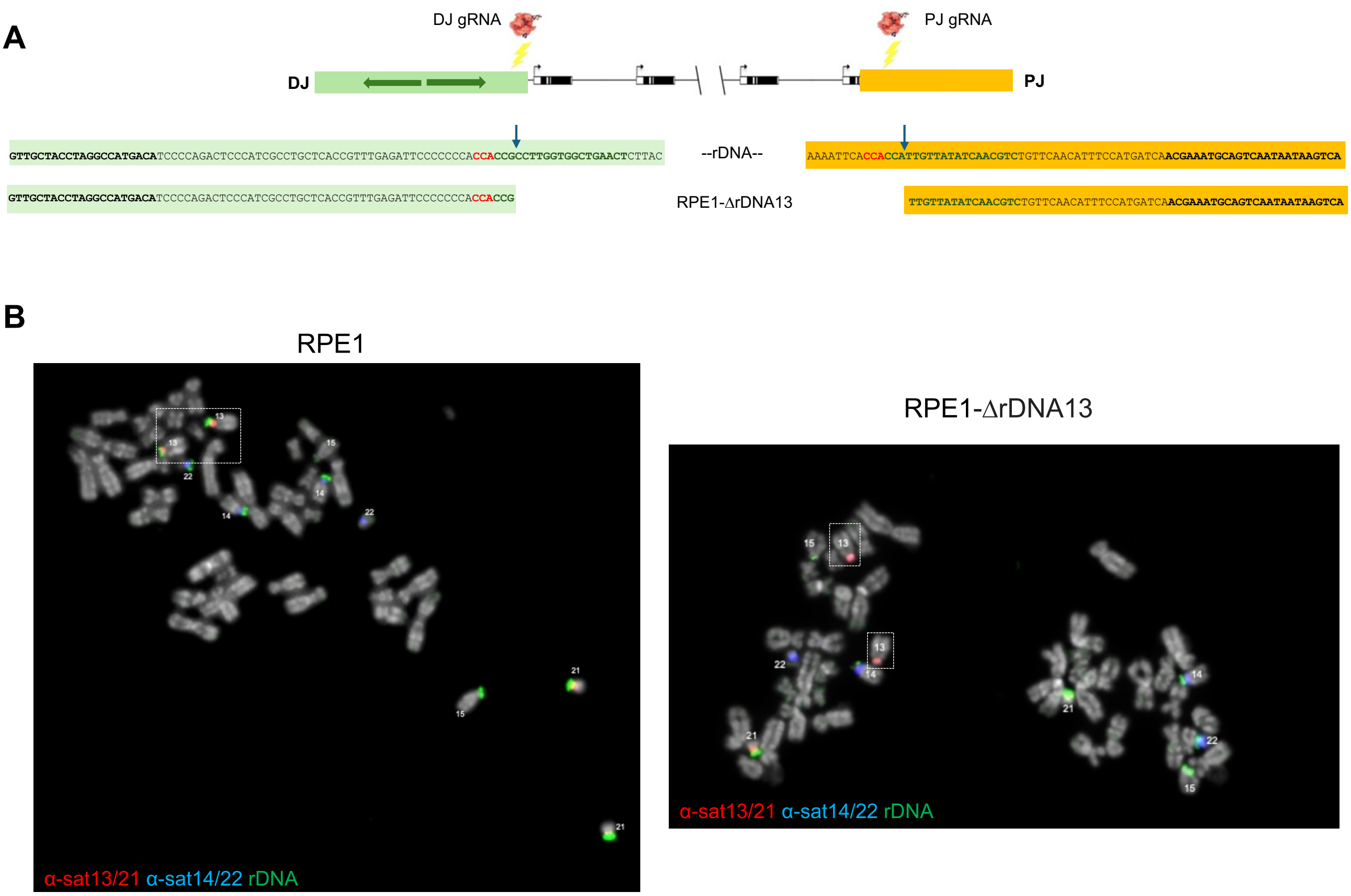

Supplemental Fig. S3 Deletion of rDNA arrays from both chr13s in RPE1 cells to generate RPE1-ΔrDNA13. (A) The sequences surrounding both DJ and PJ gRNAs are shown. The screening primers (bold) are at the start and end of the DJ and PJ sequences respectively. gRNA sequences (green), PAM sites (red) and Cas9 cleavage sites (arrow) are indicated. The fusion product, as determined by sequencing is shown below, 8 independent cloned PCR products were sequenced and they all showed the same junction, indicating that both chr13 haplotypes underwent a precise DJ/PJ fusion with no indel other than precise deletion of the rDNA. (B) M-FISH was performed on normal RPE1 cells and RPE1-ΔrDNA13 with 13/21 α-sat, 14/22 α-sat and rDNA FISH probes. Both chr13s are highlighted with dashed white line boxes and shown enlarged in Fig. 3.

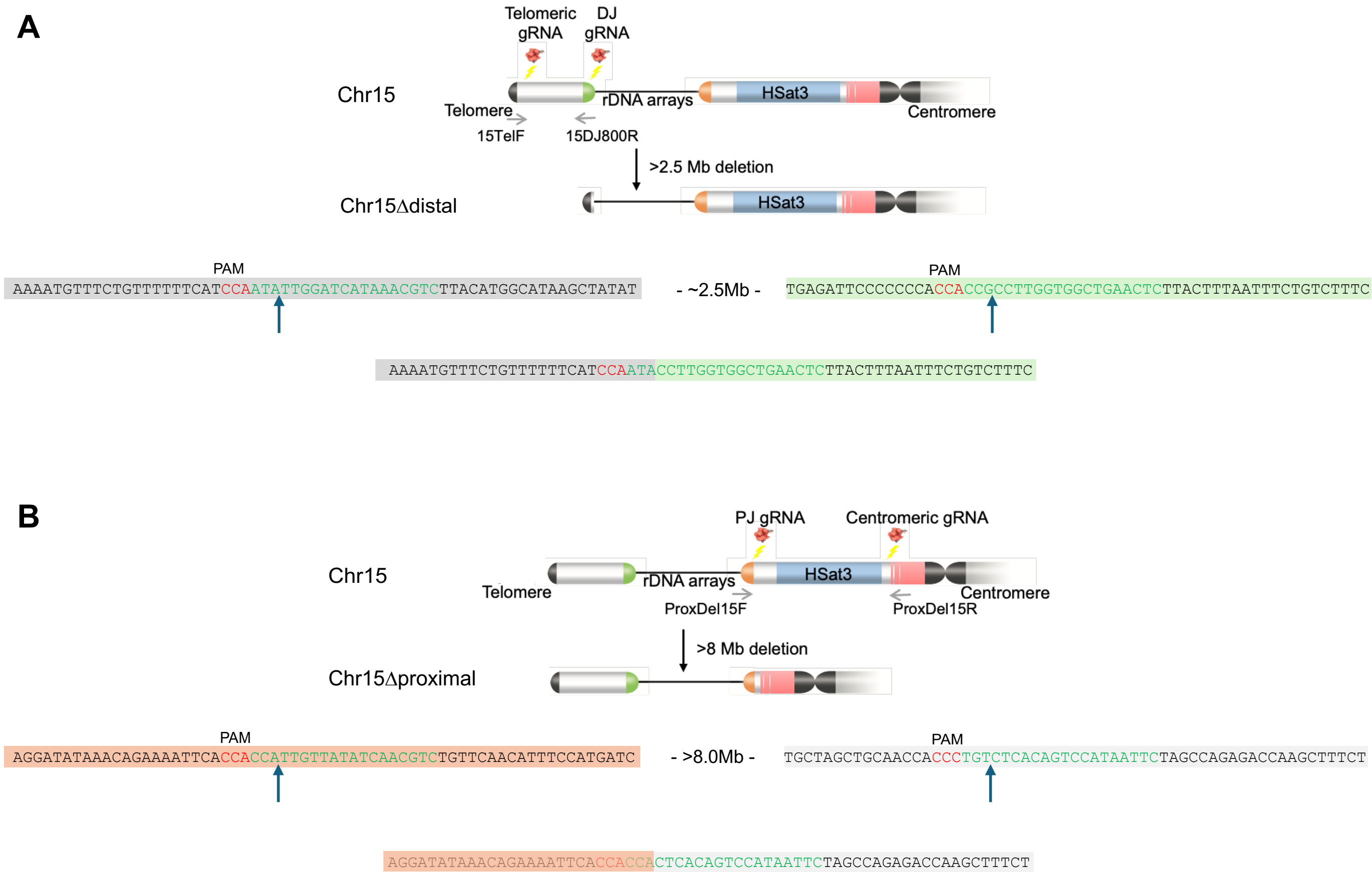

Supplemental Fig. S4 Generation of RPE1-15Δdistal and RPE1-15Δproximal cell lines. (A) Cartoon showing the CRISPR-mediated distal deletion strategy, the relative location of gRNAs and screening primers. The sequences surrounding both the 15 telomeric and DJ gRNAs are shown below. gRNA sequences (green), PAM sites (red) and Cas9 cleavage sites (arrow) are indicated. The fusion product, as determined by sequencing is shown below, indicating that a precise Telomere/DJ fusion had occurred. (B) Cartoon showing the CRISPR-mediated proximal deletion strategy, the relative location of gRNAs and screening primers. The sequences surrounding both the PJ and 15 centromeric gRNAs are shown below. gRNA sequences (green), PAM sites (red) and Cas9 cleavage sites (arrow) are indicated. The fusion product, as determined by sequencing is shown below, indicating that a precise PJ/centromere fusion had occurred.

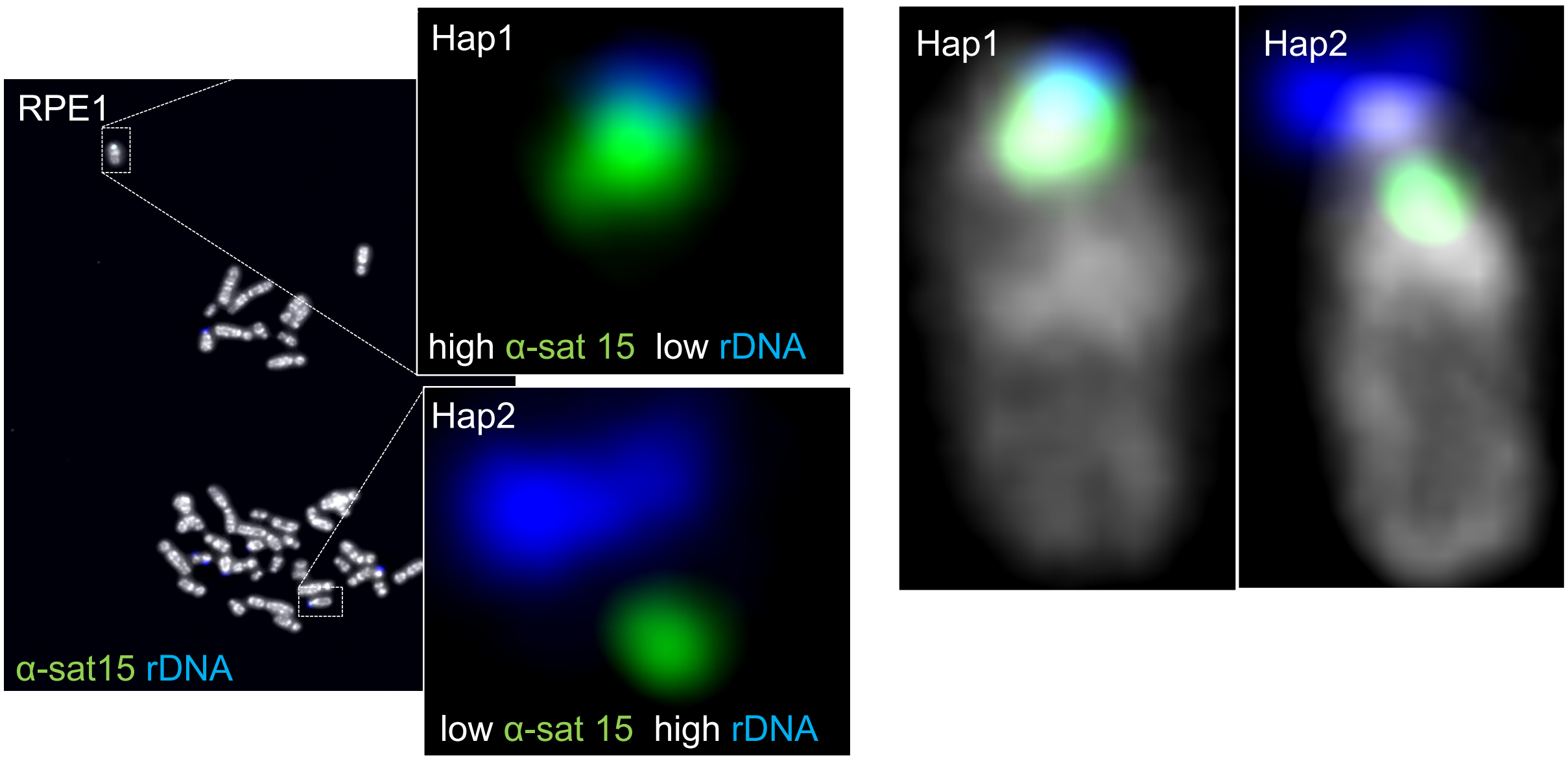

Supplemental Fig. S5 Distinguishing Chr15 haplotypes in RPE1 cells. M-FISH performed on RPE1 metaphase spreads with 15 α-sat and rDNA FISH probes. Hap1 is distinguished by having high α-sat and low rDNA content. In contrast, Hap2 is distinguished by having low α-sat and high rDNA content. The full spread is shown on the left, enlarged chr15 Hap1 and Hap2 without and with DAPI in the centre and right panels respectively.

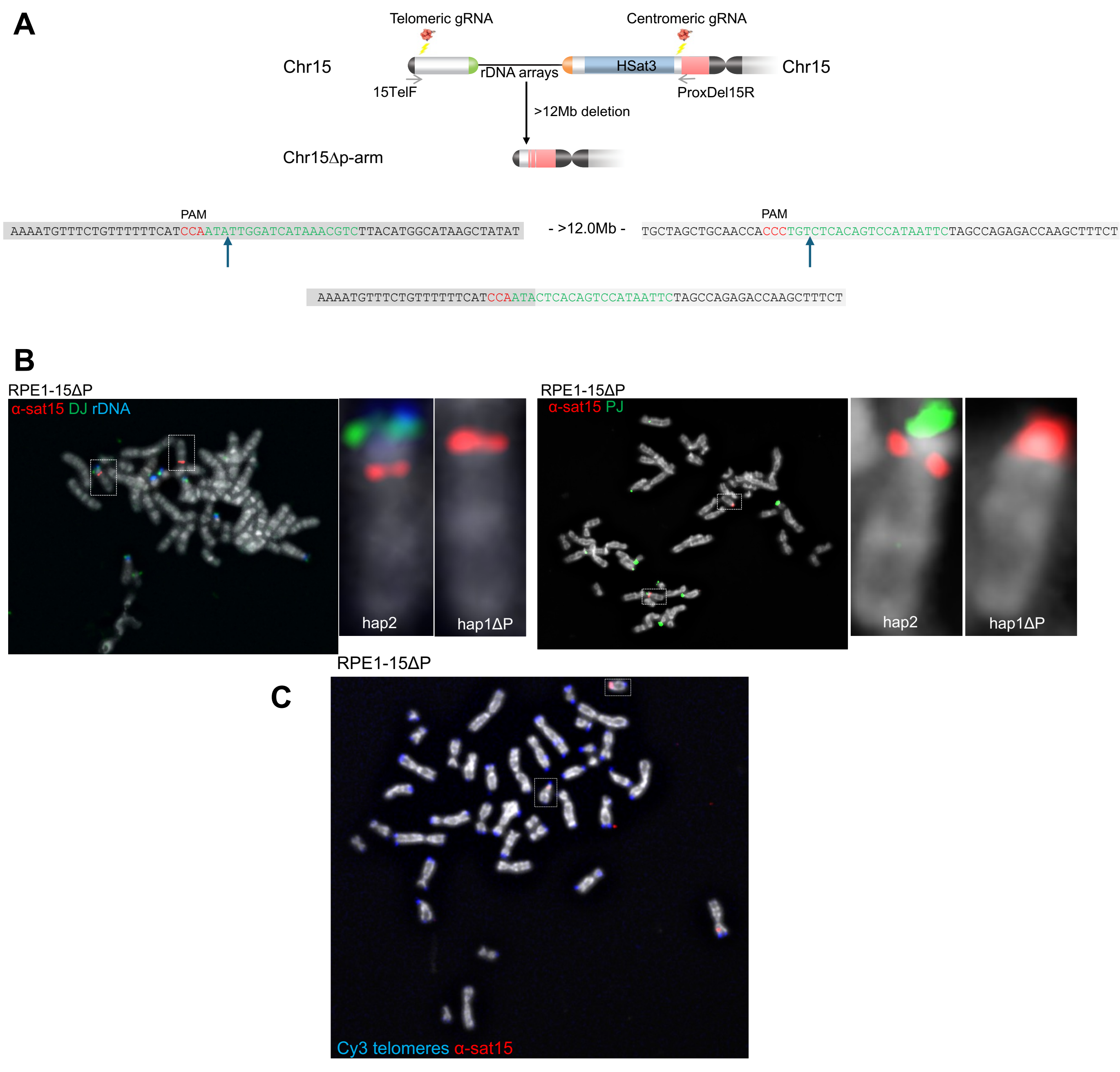

Supplemental Fig. S6 Generation of RPE1-15ΔP cells. (A) Cartoon showing the CRISPR-mediated distal deletion strategy, the relative location of gRNAs and screening primers. The sequences surrounding both the 15 telomeric and 15 centromeric gRNAs are shown below. gRNA sequences (green), PAM sites (red) and Cas9 cleavage sites (arrow) are indicated. The fusion product, as determined by sequencing, is shown below, indicating that a precise telomere/centromere fusion had occurred. (B) M-FISH on spreads from RPE1-15ΔP cells with α-sat15, rDNA and DJ FISH probes (left panels) or α-sat15, rDNA and PJ FISH probes (right panels). Enlarged versions of both 15 haplotypes are shown to the right of full spreads. These confirm the p-arm has been deleted from chr15 haplotype 1. (C) FISH on RPE1-15ΔP metaphase spreads with a telomere (Cy3 labelled) and α-sat15 probes. A full metaphase spread is shown, enlarged versions of both chr15 haplotypes are shown in Fig. 5.

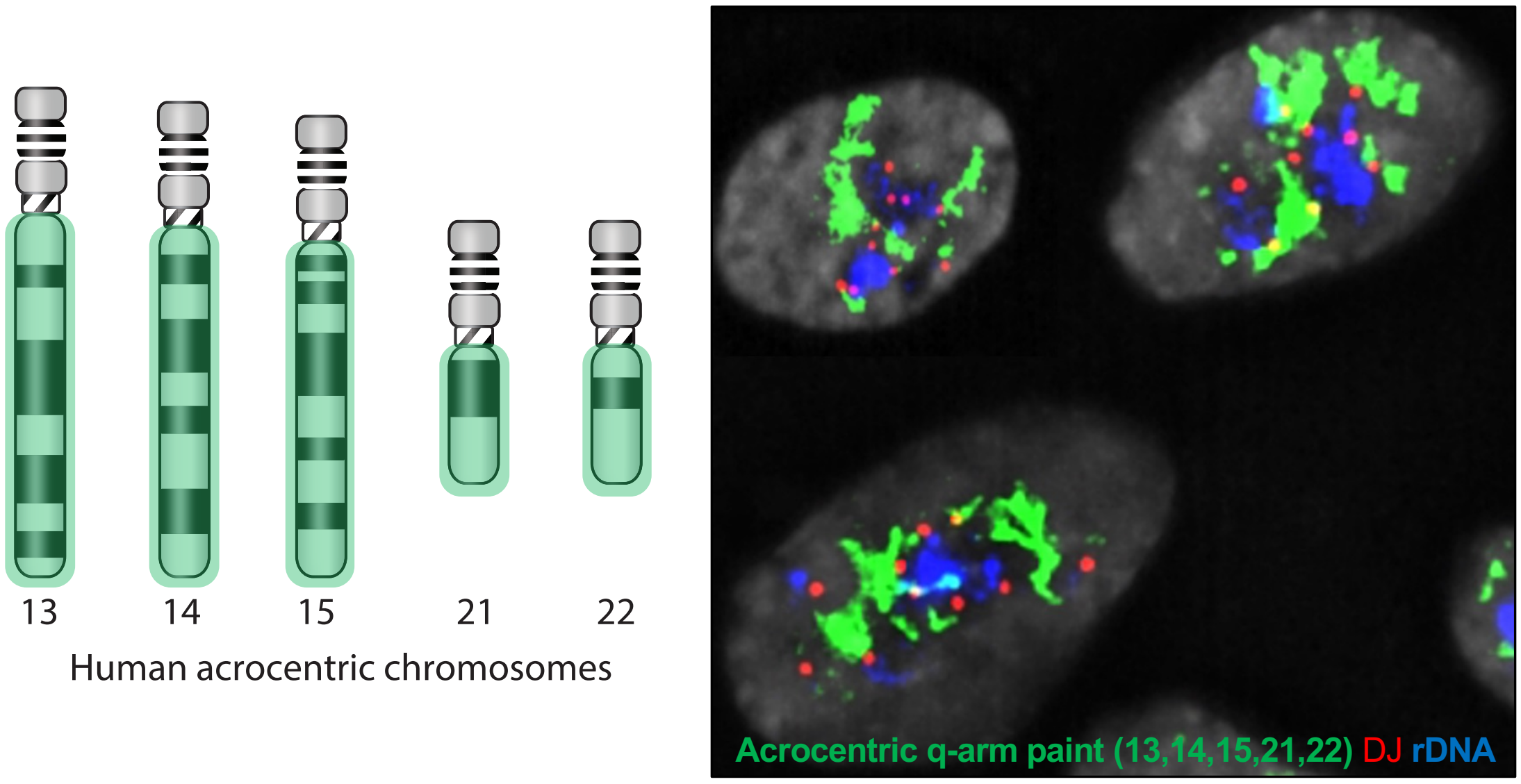

Supplemental Fig. S7 Acrocentric q-arms cluster around nucleoli in RPE1 cells. 3D-immunoFISH performed on RPE1 cells using pooled acrocentric q-arm paints (illustrated on the left) and a DJ FISH probe combined with Nop52 antibodies.

**Oligonucleotides (supplied by Integrated DNA Technologies)**		
**PCR primers used in gRNA synthesis**		
PJ gRNA (T7PJEX)	GGATCCTAATACGACTCACTATAGGACGTTGATATAACAATGGGTTTAAGAGCTATGCTG	
DJ gRNA (T7DJ804EX)	GGATCCTAATACGACTCACTATAGAGTTCAGCCACCAAGGCGGGTTTAAGAGCTATGCTG	
15 centromere gRNA (T7 ProxDel15)	GGATCCTAATACGACTCACTATAGAATTATGGACTGTGAGACAGTTTAAGAGCTATGCTG	
15 telomeric gRNA (15TEL)	GGATCCTAATACGACTCACTATAGGACGTTTATGACCCAATATGTTTAAGAGCTATGCTG	
Scaffold sequence	GTTTAAGAGCTATGCTGGAAACAGCATAGCAAGTTTAAATAAGGCTAGTCCGTTATCAACTTGAAAAAGTGGCACCGAGTCGGTGCTTTT	
T7gRNArev	AAAAGCACCGACTCGGTGCC	
**Standard PCR primers**		
novel DJ/PJ junction for deletions of rDNA in A9 hybrids and RPE1 cells	F TGACTTATTATTGACTGCATTTCGT<br>R GTTGCTACCTAGGCCATGACA	
novel junction for proximal deletion	F TACCGTATAACATTTCAAACTGGG<br>R TCTCAGTGCCTGGAGGATGTA	
novel junction for distal deletion	F TGATGATGCGAGATGGGCTG<br>R TGGTGGTGATGAAAGAGGCA	
novel junction for p-arm deletion	F TGATGATGCGAGATGGGCTG<br>R TCTCAGTGCCTGGAGGATGTA	
PentaE (15 STR)	F GCTACTCTGGAGGCTGAAACA<br>R TGGGTTATTAATTGAGAAAACTCCTTACAATTT	used in STR analysis of chr15s
**PCR primers for making p-arm FISH probes**		
β-sat	F GATCAGTGCAGAGATATGTC<br>R CTTGTCTAGGCTCTGCCTAC	
hSat1	F CATGAAATATCAAAGTTCACAAACC<br>R GGGTACATTGATATTATATGTACAG	
hSat3	F CATTCGAGTCCATTCCTCTC<br>R GAAACGAGTGGAATGGAATG	
α-satellite primers	α-SatF GAAGCTTA(A/T)(C/G)T(C/A)ACAGAGTT(G/T)AA<br>α-SatR GCTGCAGATC(A/C)C(A/C)AAG(A/T/C)AGTTTC	
**Primers for chromosome paints**		
Inter-Alu primers	F GGATTACAGG(C/T)(A/G)TGAG*C*C*A<br>R (A/G)CCA(C/T)TGCACTCCAGC*C*T*G	*phosphorothioate (Liu et al. 1993)
**BAC and plasmid FISH probes**		
DJ	BAC clone CH507-535F5 (GenBank: CT476834)	
PJ	BAC clone bP-2154M18 (GenBank: CR381535)	
Human rDNA	pUC-hrDNA-12.0 (Addgene #206320)	
q-arm 13 BAC	BAC clone RP11-420H1 (GenBank: AC018739)	
q-arm 14 BAC	BAC clone RP11-115A12 (GenBank: AC010766)	
q-arm 15 BAC	BAC clone RP11-32B5 (GenBank: AC068446)	
q-arm 21 BAC	BAC clone RPCI-11-89H21 (GenBank: AZ517782)	
q-arm 22 BAC	BAC clone RP11-278E23 (GenBank: AC013360)	
**Peptide nucleic acid (PNA) oligonucleotides (supplied by Eurogentec)**		
Cy3-labeled C-rich telomere probe	PN-TC050-005	

Supplemental Table S1

Antibodies		
Nop52 (sheep)	McStay lab	N/A
UBF (sheep)	McStay lab	N/A
TREACLE (sheep)	McStay lab	N/A
NUCLEOLIN (sheep)	McStay lab	N/A
Anti-sheep Alexa Flour® 488	JacksonImmuno Research (JIR)	Cat#713-545-147
Anti-sheep Alexa Flour® 594	JIR	Cat#713-585-147
Anti-sheep Alexa Flour® 647	JIR	Cat#713-605-147
Anti-sheep DyLight™ 405	JIR	Cat#713-475-147

Supplemental Table S2
